# Genome Sequences of RIGVIR Oncolytic Virotherapy Virus and Five Other Echovirus 7 Isolates

**DOI:** 10.1128/genomeA.00317-18

**Published:** 2018-04-26

**Authors:** Eero Hietanen, Teemu Smura, Marika Hakanen, Jira Chansaenroj, Pirjo Merilahti, Janne Nevalainen, Sunita Pandey, Satu Koskinen, Lav Tripathi, Yong Poovorawan, Juha Pursiheimo, Petri Susi

**Affiliations:** a Institute of Biomedicine, University of Turku, Turku, Finland; b Medicum, University of Helsinki, Helsinki, Finland; c Center of Excellence in Clinical Virology, Department of Paediatrics, Chulalongkorn University, Bangkok, Thailand; d Turku University Hospital, Turku, Finland

## Abstract

We report here the nearly complete Illumina-sequenced consensus genome sequences of six isolates of echovirus 7 (E7), including oncolytic virotherapy virus RIGVIR and the Wallace prototype. Amino acid identities within the coding region were highly conserved across all isolates, ranging from 95.31% to 99.73%.

## GENOME ANNOUNCEMENT

Echovirus 7 (E7) belongs to the Enterovirus *B* (EV-B) species (genus Enterovirus, family Picornaviridae), which is phylogenetically distinct from the other enterovirus species (1, 2). E7 is commonly detected in epidemiological surveys (3–5). Interestingly, E7 is one of the few enteroviruses used in oncolytic virotherapy (6). A drug virus preparation called RIGVIR is a cell-adapted E7 which has been approved for clinical use in Latvia, and it is also made available for the treatment of melanoma in other countries (7). RIGVIR is claimed to be effective against many types of cancer. However, molecular data about RIGVIR are limited. This report describes the near-complete genome sequences of RIGVIR, E7 Wallace prototype (from ATCC), and four clinical E7 isolates from Finland (98-57213, 98-59065, 98-60628, and 07VI447).

Viral RNA was extracted from virus-infected rhabdomyosarcoma (RD) cell lysates or, in the case of RIGVIR, directly from the drug virus ampule using the E.Z.N.A. viral RNA (vRNA) kit (Omega Bio-tek). Viral RNA was prepared for sequencing using the NEBNext Ultra RNA library preparation kit (catalog number E7530, New England BioLabs) and NEBNext multiplex oligos for Illumina adapter kit (catalog number E7335) and sequenced on the Illumina MiSeq platform. Contigs were assembled using *de novo* assembly protocols (MIRA version 4.0.2). Sequence and phylogenetic analyses were conducted using BioEdit (version 7.2.5) and MEGA (version 7) (8, 9).

At present, there are eight Illumina-sequenced and four Sanger-sequenced near-full-length E7 genome sequences in GenBank (as of 14 February 2018). Sanger-sequenced E7 Wallace (GenBank accession number AY302559) (1) and UMMC (GenBank accession number AY036578) (10) genomes were used as reference sequences in this study.

The nearly complete genomes, excluding the 3ʹ-poly(A) tail, of RIGVIR, the Wallace prototype, and four Finnish E7 isolates, 98-57213, 98-59065, 98-60628, and 07VI447, included in this study were 7,410 nucleotides (nt), 7,405 nt, 7,390 nt, 7,426 nt, 7,402 nt, and 7,425 nt in length, respectively. The sequenced genomes contain a 5ʹ untranslated region (UTR) (742 nt, 731 nt, 719 nt, 740 nt, 729 nt, and 741 nt in length, respectively) and a 3ʹ UTR (83 nt, 89 nt, 87 nt, 101 nt, 88 nt, and 99 nt in length, respectively). Each genome possessed a single large open reading frame (ORF) of 6,582 nt encoding a polyprotein of 2,193 amino acids in length. Comparing the sequences to that of the prototype Wallace (GenBank accession number AY302559), the whole-genome nucleotide sequence identities ranged from 79.43% to 99.81%. Within the ORFs, the nucleotide identities with respect to the Wallace prototype (GenBank accession number AY302559) ranged from 78.86% to 99.85%, whereas the amino acid identities were highly conserved, ranging from 95.31% to 99.73%. All of the sequences had similar G+C contents ranging from 47.36% to 48.04%. Sequence and phylogenetic analyses revealed that the prototype Wallace (originally isolated in the 1950s) was most closely related to 98-57213, while E7 isolates 98-59065 and 98-60628 (isolated in the 1990s) formed their own phylogenetic subtree with RIGVIR. The E7 isolate 07VI447 (isolated in 2007) was an outlier from the recent E7 isolates, being most closely related to the UMMC reference sequence (GenBank accession number AY036578).

### Accession number(s).

The complete genome sequences of E7 isolates have been deposited in GenBank under the accession numbers MH043132 (Wallace), MH043133 (98-57213), MH043134 (98-59065), MH043135 (98-60628), MH043136 (07VI447), and MH043137 (RIGVIR).
